# Insight Into the Prospects for the Improvement of Seed Starch in Legume—A Review

**DOI:** 10.3389/fpls.2019.01213

**Published:** 2019-10-31

**Authors:** Rupesh Tayade, Krishnanand P. Kulkarni, Hyun Jo, Jong Tae Song, Jeong-Dong Lee

**Affiliations:** School of Applied Biosciences, Kyungpook National University, Daegu, South Korea

**Keywords:** legumes, carbohydrate, starch, food, breeding

## Abstract

In addition to proteins and/or oils, mature seeds of most legume crops contain important carbohydrate components, including starches and sugars. Starch is also an essential nutritional component of human and animal diets and has various food and non-food industrial applications. Starch is a primary insoluble polymeric carbohydrate produced by higher plants and consists of amylose and amylopectin as a major fraction. Legume seeds are an affordable source of not only protein but also the starch, which has an advantage of being resistant starch compared with cereal, root, and tuber starch. For these reasons, legume seeds form a good source of resistant starch-rich healthy food with a high protein content and can be utilized in various food applications. The genetics and molecular details of starch and other carbohydrate components are well studied in cereal crops but have received little attention in legumes. In order to improve legume starch content, quality, and quantity, it is necessary to understand the genetic and molecular factors regulating carbohydrate metabolism in legume crops. In this review, we assessed the current literature reporting the genetic and molecular basis of legume carbohydrate components, primarily focused on seed starch content. We provided an overview of starch biosynthesis in the heterotrophic organs, the chemical composition of major consumable legumes, the factors influencing starch digestibility, and advances in the genetic, transcriptomic, and metabolomic studies in important legume crops. Further, we discussed breeding and biotechnological approaches for the improvement of the starch composition in major legume crops. The information reviewed in this study will be helpful in facilitating the food and non-food applications of legume starch and provide economic benefits to farmers and industries.

## Introduction

Legumes belong to Fabaceae, the third largest and economically important plant family, which has more than 20,000 species including herbs, shrubs, and trees (Doyle, 2003; Gepts et al., 2005; Lewis, 2005; Dash et al., 2016). From these legumes, a limited number of species including lentils, beans, peas, pulses, and soybeans are used as human food and animal feed. Legumes are a healthy and affordable source of protein and provide excellent nutritional support to humans and animals. The average protein content in legume crops (20–40%) is two to three times that of wheat (*Triticum aestivum*) and rice (*Oryza sativa*) (Reyes-Moreno and Paredes-López, 1993). For instance, cowpea (*Vigna unguiculata*) contains ∼25% protein (Bressani, 1985; Annor et al., 2010), whereas soybean contains ∼40% protein in their mature seeds (Burton, 1997). In general, legumes have a low glycemic index (GI) value and high content of dietary fibers, polyunsaturated fatty acids, and complex carbohydrates, in addition to small amounts of minerals such as magnesium, potassium, essential vitamins, and phytochemicals (Campos-Vega et al., 2010; Rebello et al., 2014). In addition, several legume seeds contain beneficial bioactive compounds with antioxidant properties, which make them a better source of functional foods. Such functional foods may be helpful in the prevention of several diseases such as cancers, heart or cardiovascular diseases, osteoporosis, and some degenerative diseases (Campos-Vega et al., 2010; Rebello et al., 2014).

Although most of the legumes are a rich source of protein, some legumes are also vital sources of carbohydrates (Jood et al., 1985). In order to suffice required energy demands growth and regrowth, plants accumulate insoluble and soluble carbohydrates in storage forms (pectin, cellulose, hemicellulose, polysaccharides, starch and glucose, fructose, sucrose, raffinose, stachyose, and verbascose). Carbohydrates are an essential component of legume seeds, and their composition varies among species. Commonly, carbohydrates are composed of 65–72% starch and 10–20% dietary fiber (Pehrsson et al., 2013). Starch is an insoluble polymeric carbohydrate produced by most of the higher plants, which consists of amylopectin and amylose. Starch is the main component of carbohydrates in human diets (Mahadevamma et al., 2004), and also essential in various food and non-food industrial applications such as adhesives, paper, textiles, biodegradable plastics (Jobling, 2004; Mooney, 2009), and bioethanol production (Goldemberg, 2007). Since starch is the major component of most legume carbohydrates, the scientific community has been trying to address the question of how legumes can be an excellent food source of dietary fiber. Until the 1980s, it was commonly believed that all starch ingested by humans instantly hydrolyzes to glucose. However, Englyst and Cummings (1987) showed that not all starch is immediately digested into glucose in the small intestine and that some of it remains encapsulated intact in the cell. This undigested encapsulated starch remains isolated to digestive enzymes and is later transferred to the large intestine where it is fermented by the microorganisms to subsequently produce important metabolites (Englyst et al., 1992; Dhital et al., 2016). Commonly, starch not digested by amylases is known as “resistant starch” (RS), and it was confirmed that RS functions much like dietary fiber in food (Birt et al., 2013). The soluble and insoluble types of dietary fiber mostly available in food are different in composition and solubility in gastrointestinal fluid. The insoluble fiber utilized by gut bacteria acts as a bulking agent and helps in the digestion process, whereas the dietary fiber soluble in the gastrointestinal fluid produces thick and viscous honey-like syrup. The soluble fiber is metabolized by the gut bacteria, which convert much of it into smaller molecules called short-chain fatty acids and help maintain the health of colonic cells (Topping and Clifton, 2001). Since the mobilization of RS does not occur in the small intestine, it reduces glucose levels in the blood, thereby minimizing insulin requirements (Behall and Howe, 1995). Hence, foods containing RS increase satiety with low GI and are beneficial to humans.

The cereals are important sources of calories in the human diet and provide feed for animals. They are worthy sources of starch. Commonly, starch is the major component of cereal grains, which consists of 60–80% starch. Foods from high-starch cereals such as maize (65–75%), wheat (60–75%), rice (80%), and potato (*Solanum tuberosum* L.) (7–20%) have very limited RS but release more glucose in the blood (Rahen et al., 1994). Conversely, foods from legumes are a greater source of RS (20–30%) on the basis on g/100 g dry matter, half of which is resistant to digestion (Bednar et al., 2001). However, seed starch levels widely vary across legumes. For instance, the seed starch content (SSC) in pea (*Pisum sativum* L.) is about 50% of its dry weight, whereas the SSC is very low in soybean (≤0.91%) (Wilson et al., 1978) and *Medicago truncatula* (≤1–2%) (Song et al., 2017). However, recently, Dhungana et al. (2017a) evaluated the soybean (*n* = 17) genotypes with varied starch concentration. The result indicated that the highest value of starch content was from IT183905 genotype with 1.4%, and the least starch content was from IT228277 with 0.24%. Previously, Koo et al. (2014) used a starch–iodine test to identify varieties with high starch content in 2,354 soybean germplasm and reported 2.81∼4.55% starch contents in seven soybean germplasms. However, starch–iodine test may give high estimation if the starch is high in amylose content or enriched with amylopectin. The wide range of variation of starch levels in legumes is mostly due to varying levels of starch synthesis in the seeds or degradation during seed maturity (Gallardo et al., 2008). Several Asian and African countries prepare various food recipes after the appropriate processing of legumes. In particular, soybeans traditionally have been used to prepare different kinds of fermented or non-fermented foods and are also consumed directly. Cooked soybean starch imparts sweetness and softness to the food product (Masuda, 2004; Jeong et al., 2010). Consequently, soybeans with high starch content, which varies with growth stages, could be beneficial in various food products such as boiled soy sprouts, fermented soybean paste, tempeh, miso, natto, and chungkoojang (Ghani et al., 2016).

In recent years, there have been steady increases in the production and consumption of legumes, mainly due to increased awareness about the nutritional and health benefits of legume-based food products. Soybeans differ from other widely grown legume crops, as they have high protein content, which, in combination with oil, forms an average of 60% of the total seed content. Although the expression of carbohydrate and starch biosynthesis genes in soybean seeds was continuously increased before full seed stage, it was later reduced significantly than genes related to fatty acid and oil synthesis (Yang et al., 2015). Likewise, the expression of starch biosynthesis genes was continuously increased at the matured seed stage in the adzuki bean, a high starch legume crop, whereas the expression of genes related to fatty acid biosynthesis in the adzuki bean was decreased at the later seed stage (Yang et al., 2015). It is expected, therefore, that several starch-metabolizing enzymes might be actively involved in the process of conversion of starch to protein or oil in seeds during seed maturation (Yang et al., 2015). At present, the genetic and molecular details of these mechanisms are still elusive, particularly in soybean. Thus, understanding the metabolism and biosynthesis pathways involved in starch synthesis and degradation in legume plants will promote crop improvement in starch for food, non-food, nutritional, and health applications. In this article, we provide an overview of starch biosynthesis in legume crops. Briefly, we discuss recent advances in genetic, transcriptomic, and metabolomic efforts in legume crops. We further analyze the factors influencing the RS content and its genetic variability, and we present the breeding and biotechnological approaches useful for developing legume cultivars with improved seed starch compositions.

## Significance of Legume and Starch in Human Diet

In recent decades, increased awareness of the use of legume seeds as source of nutritional food and animal feed is of the highest importance for the development of legume crops. The legume or pulse family has nutritional compositions, and health benefits have been a hot topic in recent decades (Chibbar et al., 2010; McCrory et al., 2010). For instance, the common bean has several beneficial biological activities that provide antioxidant effects, reduce cholesterol and lipoprotein, and have anti-mutagenic and anticancer effects as well as effects on cardiovascular diseases, diabetes, and obesity (Suárez-Martínez et al., 2016).

Commonly, the prime nutritional fraction of carbohydrate is starch in legume seeds. Generally, amylose and amylopectin comprise about 98–99% of the dry weight of total starch. Amylose content as well as long chains of amylopectin extends the retrogradation and increases benefit in reducing the glucose released in the blood, exhibits prebiotic effects beneficial for colonic microflora, and eventually promotes a lower GI. It is well known that processing and cooking methods with legume crops produce an increase in the levels of RS, which has slow digestibility resulting in the positive impact of GI. It leads to better benefits in human diet to control type 2 diabetes (reviewed by Sajilata et al., 2006; Birt et al., 2013; Patto et al., 2015).

In general, the RS content of legumes has been reported to be higher than that of cereals and tubers crops as several studies have investigated. However, literature reports varied regarding legume RS content between the species in the reported studies. For instance, Yadav et al. (2010) reported as low as 3.4% RS content in pea, as high as 4.9% RS content on the dry weight basis in lentils. Earlier, Murphy et al. (2008) investigated the RS concentration from the database. They reported the values of RS content for various food sources on the basis of g RS per 100 g of food. For instance, cooked cowpeas have 0.6% RS content, cooked lentils 3.4% RS content, mature/cooked/canned peas 2.6% RS content, cooked/canned kidney beans 2.0% RS content, and cooked/canned white beans 4.2% RS content. The reports on the variation in the RS contents of legume influence by processing are shown in **Table 1**. The expected daily intake of RS for Americans is recommended to be a minimum 6-g intake of RS in the daily diet for health perspective (Murphy et al., 2008). However, intake estimation differs between nations (Lockyer et al., 2016). Several studies based on many rodents have demonstrated that RS has numerous metabolic health benefits. For instance, RS plays a vital role in improving significant animal gut health: it can improve insulin sensitivity, increase gastrointestinal tract incretin hormone, increase glucagon-like peptide 1 (GLP-1), decrease body fat, and reduce overweight (Keenan et al., 2015). However, the mechanisms for this health benefit remain elusive. The RS content varies between species that are of great interest for their nutritional products for humans. Legume starch has been used in various food industrial applications. Due to its gelling, emulsion, and stabilizing capabilities, it has been used in soups and bread to improve the thickness and texture, to replace fat, to enhance the crispiness of food product, and to improve the mouthfeel of yoghurt (Messina, 1999; Mlyneková et al., 2014; Ndidi et al., 2014).

**Table 1:** Chemical compositions of major legume seeds with amylose content and resistant starch.

Crop	Crude protein(%)	Crude fiber(%)	Fat(%)	Starch(%)	Amylose(%)	Raw RS^b^(%)	Processed RS^b^(%)	Ash(%)	References
Adzuki bean	24.0	7.0	6.0	48	19.2	26.3	–	3.9	Tjahjadi and Breene, 1984; Shi et al., 2017; Reddy et al., 2017
Black gram	24.6	7.2	1.3	24.4	40.6	11.4	50.3	4.1	Kaur and Kapoor, 1991; Sandhu and Lim, 2008; Wani et al., 2015
Chickpea	22.8	3.5	5.4	50.4	13.6	3.4	51.4	3	Wood and Grusak, 2007; Qayyum et al., 2012; Kasote et al., 2014; Sundell et al., 2017
Common bean	25.4	17.4	1.7	37.4	51.1	3.7	2.3	3.8	De Almeida Costa et al., 2006; Marquezi et al., 2016; Suárez-Martínez et al., 2016
Cowpea	28.0	3.1	1.9	40.6	42.7	9.6	–	3.8	Longe, 1980; Hoover and Ratnayake, 2002; De Almeida Costa et al., 2006; Ratnaningsih et al., 2016; Eshwarage et al., 2017
Lentil	24.6^a^	10.7^a^	1.1^a^	49.9^a^	24.7	3.2	50.3	2.71^a^	Hoover and Ratnayake, 2002; Sandhu and Lim, 2008
Lotus	4.1^a^	–	0.5^a^	–	30.6	–	–	1.1^a^	Geng et al., 2007
Mung bean	23.9	3.9	1.2^a^	45.0	31.1	11.6	50.2	3.7	Sandhu and Lim, 2008; Hedley, 2000; Kasote et al., 2014
Navy bean	22.7	4.2	0.6^a^	15.4	28.6	4.2	–	4.1	Hoover and Ratnayake, 2002
Pea	23.9	9.2	1.6	43.4	88.0	2.4-	52.5	3.3	Ratnayake et al., 2002; Zhou et al., 2004; De Almeida Costa et al., 2006; Sandhu and Lim, 2008; Červenski et al., 2017
Peanut	25.2	2.1	49.7^a^	11.5	–	–	–	2.3	Jambunathan, 1991
Pigeon pea	21.0	2.5	1.7	57.5	28.4	16.9	50.9	3.5	Singh et al., 1993; Sandhu and Lim, 2008; Eltayeb, 2010; Kasote et al., 2014
Pinto bean	5.3	3	0.9	30.1	37.4	35.5	–	1.0	Hoover and Ratnayake, 2002; Zhou et al., 2004; Fabbri et al., 2016
Soybean	40	1.5	21	0.9	16.2	0.1	–	5	Wilson et al., 1978; Stevenson et al., 2006
White lupin	30.6	5.2	14.6	3.3	–	–	–	4.0	Jul et al., 2003; Kohajdova et al., 2011
Yellow lupin	37.9	4.9	8.7	4.5	–	–	–	6	Jul et al., 2003; Kohajdova et al., 2011

a Nutrient Data Laboratory, ARS, USDA National Food and Nutrient Analysis Program Wave 6m, 2002 Beltsville MD.

b RS, resistant starch.

Recently, overconsumption of starch or carbohydrates increases the risk of diabetes and other cardiovascular diseases, which appeals for the change of additional digestion-resistant and reduced GI legume crop or food. In addition, global starch demand is ever increasing; therefore, there is a need to look for new starch sources. Legumes are the best known source for nutritional starch, and they can provide the best option to substitute cereal or tuber starch in suitable food and industrial application.

## Starch Metabolism in Plants

**Figure 1:**
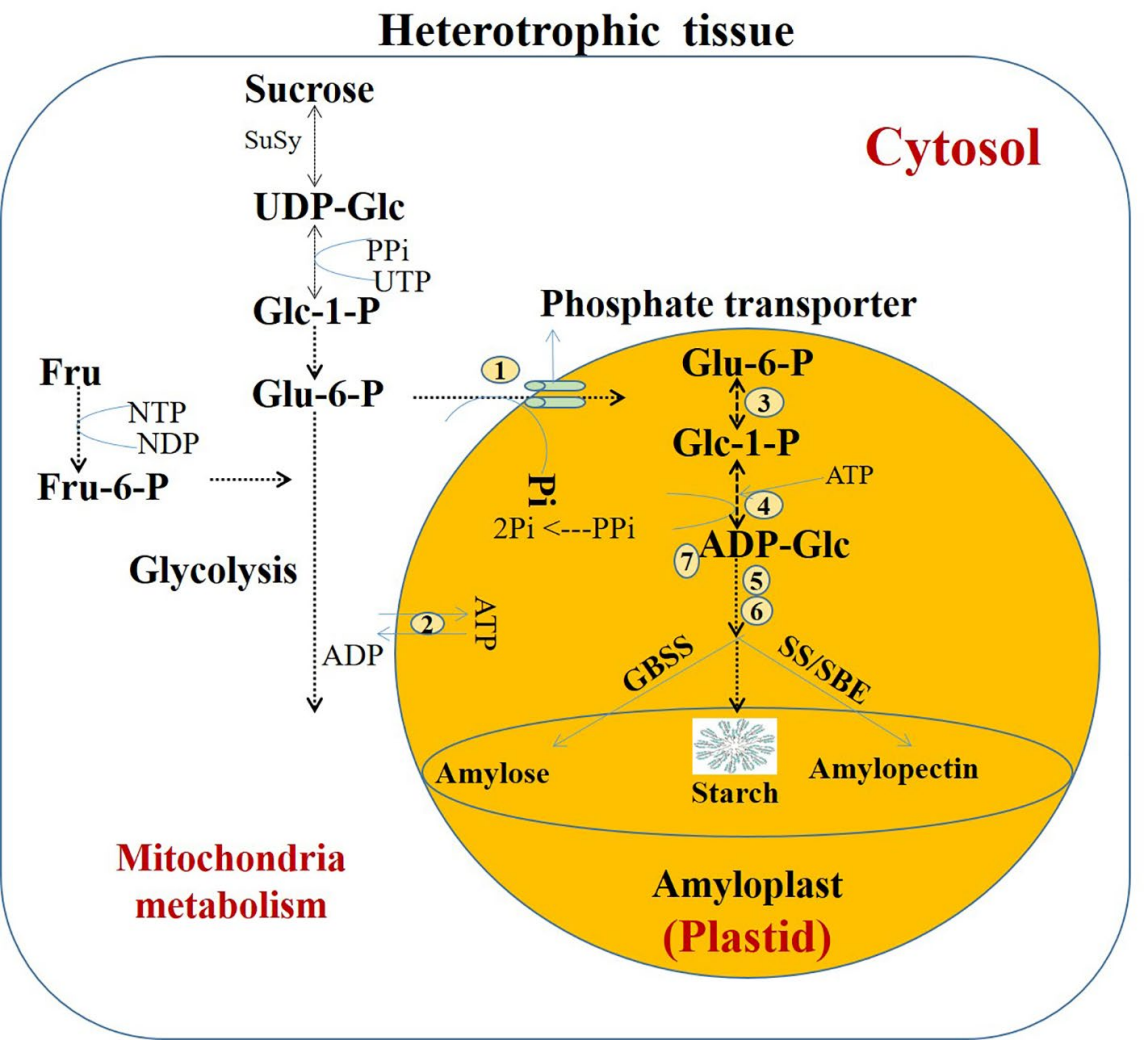
Starch metabolism in heterotrophic tissue, highlighted in the orange circle; ADP-Glucose (ADP-Glc) enters the plastid by the specific translocator, that is, a phosphate transporter and the ATP/ADP transporter. Subsequently, adenosine 5′-diphosphate glucose pyrophosphorylase (AGPase) enzyme induces the regulating reaction in amyloplasts by converting Glucose-1-phosphate (Glc-1-P) and ATP to ADP-Glc and inorganic pyrophosphate (PPi). Systematic interaction of multienzymes gradually leads to synthesized starch. The major metabolites and enzymes involved in the process: 1, glucose 6-phosphate transporter; 2, amyloplast adenylate transporter; 3, plastidial phosphoglucomutase; 4, ADP–glucose pyrophosphorylase; 5, starch synthases (SS); 6, starch branching enzymes (SBE); 7, inorganic pyrophosphatase. Sucrose synthases (SuSy), granule-bound starch synthase (GBSS), Fructose (Fru), Fructose-6-phosphate (Fru-6-P). Modified from Bahaji et al. (2014).

In higher plants, starch is broadly categorized as either transient or storage starch. Starch granules vary in size, shape, composition, and properties according to species, organ, and stage of development (Jane et al., 1994). Transient starch is synthesized in the plastids of a photosynthetic organ as a result of photo-assimilation from the Calvin cycle and utilized during the night by degradation into sucrose to provide a carbon source to the non-photosynthetic organs of the plant. In contrast, storage starch is synthesized in the non-photosynthetic tissues, such as the tuber, seeds, and roots, which require imports of sucrose (Jeong et al., 2010). Three enzymes are mainly involved in the starch synthesis in plant such as, adenosine 5′-diphosphate (ADP) glucose pyrophosphorylase (AGPase), starch synthase (SS) and starch-branching enzymes (SBEs). Starch biosynthesis in heterotrophic organs starts with the cytosolic pathway where uploaded sucrose gets converted to uridine diphosphate (UDP) into UDP-glucose (UDP-Glc) and fructose or alternatively ADP-glucose (ADP-Glc) and further metabolized to produce hexose phosphates in the cytosol. Moreover, in the dicot plants, the hexose phosphates (i.e., glucose-6-phosphate (Glu-6-P)) and ATP are transported into the amyloplast or plastid as the first substrate for the synthesis of ADP-Glc. Unlike cytosol, amyloplast is incapable of producing ATP due to lack of photosynthesis, and therefore, cytosolic ATP enters in to the amyloplast to produce ADP-Glc. The starch synthesis involves SS and the synchronized chain elongation reactions of α-(1→4)-linked glucan, branching at α-(1→6) positions, and debranching of specific branch linkages through the ADP-Glc key precursor. This ADP-Glc is brought into the plastid by the specific translocator also known as phosphate transporter and the ATP/ADP transporter (Neuhaus and Wagner, 2000) or transport protein *via* ADP counterexchange mechanism (Shannon et al., 1996). In starch biosynthesis, hexose phosphate precursors and ADP-Glc vary among species and tissues. In short, the transport of hexose phosphate takes place in exchange for molecules of orthophosphate (Pi), whereas ATP transport follows the exchange of ADP for Pi. However, starch biosynthesis functioning in the endosperms of monocot plants, for instance, cereals (rice, maize, and barley), differs with the import of ADP-Glc transfer from cytosol to amyloplast. In case of a monocot plant, the transport of ADP-Glc takes place through ADP-Glc/ADP transporter known as BT1 protein (Shannon et al., 1998; Patron et al., 2004). Subsequently, inside the plastid, conversion of Glu-6-P to glucose-1-phosphate (Glc-1-P) takes place by the catalytic reaction *via* plastidic phosphoglucomutase (PGM). In general, of starch synthesis starting enzyme, AGPase is activated by 3-phosphoglycerate and inhibited by inorganic pyrophosphate (PPi). This AGPase enzyme induces the regulating reaction in amyloplasts by converting Glc-1-P and ATP to ADP-Glc and PPi. The basic component of starch, amylose, and amylopectin synthesis occurs through the activity of three major enzymes, that is, granule-bound SS (*GBSS*), SS, and *SBE*s. In addition, interactions of pullulanase (*PUL*) and isoamylase (*ISA*) debranching enzyme play an important role to determine the complex structure of starch, and they are also involved in starch breakdown during germination. In brief, this systematic interaction of multienzymes gradually leads to synthesized starch (Neuhaus and Wagner, 2000). The major genes encoding enzymes involved in starch synthesis in the non-photosynthetic organs are shown in **Figure 1**. Different plant species has multiple forms of each enzyme involved in starch biosynthesis; because of this, differences in the composition of some gene families among the legumes, as well as other plant species, might be observed. For instance, Vriet et al. (2010) identified similar encoding genes involved in birdsfoot trefoil (*Lotus corniculatus* L.), which was reported to be involved in starch metabolism in *Arabidopsis*, with various differences in isoform. In addition, they are also observed in duplications of a number of starch metabolism genes in *Lotus japonicus*, including *AGPase large subunit isoform 2* (*APL2*), *SS II* (*SSII*), *GBSS*, *α-amylase 3* (*AMY3*), *β-amylase 3* (*BAM3*), and *cytosolic glucan phosphorylase* (*PHS2*). Similarly, Pan et al. (2009) reported two functional isoforms for GBSSI and SSII in starchy legumes, birdsfoot trefoil, cowpea, and mung bean (*Vigna radiata* (L.) Wilczek). In soybean, nonsense mutations in the putative coding region caused non-function of the *SSIIb* genes (Pan et al., 2009). Duplication of GBSSI and SSII has been observed in a number of legumes and cereal crops and may have an interdependent origin (Pan et al., 2009). Comprehensive analyses of the functions and processes involved in starch synthesis in phototropic and non-phototrophic organs have been performed by several researchers over the past two decades (Fernie et al., 2002; Zeeman et al., 2010; Bahaji et al., 2014). However, in contrast to starch synthesis, the pathway of starch degradation in heterotrophic tissues and organs of legume seeds is not well studied (Smith et al., 2005).

From the literatures, it indicates that starch synthesis in legumes is similar as in *Arabidopsis* leaves, and key enzymes involved in the biosynthetic pathways also the same as in *Arabidopsis*. However, in *Arabidopsis*, a number of enzymes metabolize the starch synthesis that is encoded by single genes, whereas legume plant species have multiple forms of each enzyme involved in starch biosynthesis, and these are encoded by multiple or duplication of the genes in legumes.

## Chemical Composition and Starch Content of Major Legume Seeds

Starches from different biological sources have different structural and polymer forms and, therefore, possess different physicochemical properties. These properties determine the function of the starch. Starch consists of amylose and amylopectin units, and their numbers and organization vary among different species. Legumes contain a 5% to 10% higher content of amylose starch than cereals, which commonly contain 25–28% amylose and 72–75% amylopectin (Gopalan, 1999). The chemical composition of important carbohydrate components, including starch in major legume crops, is given in **Table 1**. Commonly, starch is distinguished into three different types—A, B, and C—which are characterized based on the polymorph present in the starch granules and the X-ray diffraction pattern. For instance, cereals show the typical A type, tuber starch shows the B type, and legumes show the mixed pattern (C type). Starch is categorized on the basis of glucose release and its absorption in the gastrointestinal tract into rapidly digestible starch (RDS), slowly digestible starch (SDS), and RS (Englyst et al., 1992; Sajilata et al., 2006). Englyst and Cummings (1987) defined the partially RS and RS. Further, subsequently classified into RS type I to RS type V (Birt et al., 2013). Starch is ensnared in a food matrix, structurally inflexible, and unreachable to amylases known as RS type I, which is found in whole grains and legumes. RS type II is raw starch granule found in cereals, tubers, legumes, and fruits, which is resistant to gelatinization and digestive enzymes (Moongngarm, 2013). RS type III is “retrogradation,” starch that is processed by various means leading to recrystallization of single chain amylose to double helices, which are improper for enzymatic interaction with amylases leading to resistance to hydrolysis (Jane and Robyt, 1984; Sievert and Pomeranz, 1990; Witt et al., 2010). RS type IV is a chemically modified starch by etherification, oxidation, or cross-linking or adding chemical derivatives, generally present in bread and cake and resistant to enzymatic digestion (Woo and Seib, 2002; Han and BeMiller, 2007). In addition, another starch was investigated to be resistant to enzymatic digestion because of the formation of artificial complexes between amylose and lipids known as RS type V (Hasjim et al., 2010). *In vitro* studies by several researchers have determined the RDS, SDS, and RS fractions in overall legume starches (Chung et al., 2008; Sandhu and Lim, 2008; Chung et al., 2009). Legume starch consists of more SDS and RS, but less RDS. However, Sandhu and Lim (2008) performed a comparative analysis of different legumes such as black gram (*Vigna mungo*), chickpea (*Cicer arietinum* L.), mung bean, lentil, pea, and pigeon pea (*Cajanus cajan*) for RDS, SDS, and RS. The GI in this study was estimated based on the hydrolysis indices; and the RDS, SDS, and RS were estimated based on isolated starch sample (20 mg) from each legume. The lowest GI and RDS content observed therein were 44.2 GI and 4.2% RDS, respectively, in pigeon pea, whereas the highest was 10.9% RDS and 49.8 GI observed for chickpea. Similarly, mung bean had the lowest RS content, which was observed at 50.3%, and the highest RS 78.9% observed for pigeon pea, whereas the lowest SDS content was observed at 16.9% in pigeon pea and the highest at 40.0% in mung bean.

Literature showing evidence in general legume starches poses the lower digestibility and shows mixed types of X-ray diffraction. Legume starches have a high level of SDS, which makes them more attractive than starch of cereal or tuber crops. This leads to more appeals to the consumers for nutritional and diet perspective to use legume starches as foods.

## Factors Influencing Starch Digestibility and Content in Legumes

There are a number of factors that influence starch digestibility such as species identity, physicochemical properties, moisture content, and microstructural composition. However, some factors are very important in starch digestibility and are mentioned subsequently in this section. Starch digestibility is also affected by processing and storage conditions such as temperature, soaking, hydrolysis, germination, and sprouting (Ring et al., 1988). In addition, other factors such as the properties of gelatinization, starch granule size, the proportion of amylose and amylopectin content, the interaction of starch and protein, complex formation with lipid and amylose, food concentration of RS, and the fiber and absorption of some enzymes also influence starch digestibility (Asp et al., 1987). Legumes also contain soluble carbohydrate components, which are anti-nutrients, such as raffinose, stachyose, and verbascose content known as raffinose family oligosaccharides (RFOs) and or α-galactosides, which may affect the starch digestibility and GI value (Wang et al., 2003). These anti-nutritional factors are found in legume seed with 2–10 g/100 g of dry weight (Mittal et al., 2012; Muzquiz et al., 2012). Due to the non-absorption or hydrolysis nature of α-galactosidase and lack of galactosidase enzyme activity in the human digestive tract, they are unable to be digested (reviewed by Guillon and Champ, 2002; Reza et al., 2009). Recently, several experimental studies have shown that nutrient and non-nutrient components of legumes affect digestibility and human health (Sánchez-chino et al., 2015; Kouris-Blazos and Belski, 2016). In addition, legumes also are composed of many other anti-nutrients such as enzyme inhibitors (phytates, polyphenols, lectins, saponins, tannin, and goitrogens), cause flatulence, and have non-starch polysaccharides (Grela et al., 2017; Grela and Samoli, 2017; Singh et al., 2017). Literature indicates that because of chelating metal ions, other undesirable properties, and relation with starch and protein, these anti-nutrients interrupt several biochemical pathways, form a complex with starch or gastric enzymes, reduces palatability, impair mineral absorption and proteins digestibility, ultimately influence the starch digestibility in legumes (Butler, 1989; Carbonaro et al., 1996). Recently, Grela et al. (2017) reported anti-nutritional factors and antioxidant activity in some important legume species. For instance, they found the chickpea contained the highest phenols and polyphenols; in addition, lentils and yellow lupine seeds have higher antioxidant activities and are reported to have other vital health-protective compounds. These anti-nutrients have been shown to cause hypoglycemia and decrease growth rates in rats (Kakade and Evans, 1966). However, these adverse effects of anti-nutrient can be overcome by processing treatment and different cooking methodologies and can improve extractability and mineral bioavailability. In addition, moisture content and heating time considerably influence starch digestion quality and nutritive value (Mahasukhonthachat et al., 2010; Pelgrom et al., 2013). With the use of an *in vitro* dialysis system, processing treatments (fermentation, germination, pressure cooking, and roasting) have been shown to increase the rate of starch digestibility in the flours and processed germinating seedlings of chickpea, cowpea, and mung bean (*Vigna radiata*) (Urooj and Puttaraj, 1994). Similarly, Rehman and Shah, (2005) studied the different thermal heat treatments on anti-nutrients, protein, and starch digestibility of processed flour of black grams, chickpeas, lentils, and red and white kidney beans. They observed the improvement in the digestibility level with different heat treatments. In addition, increased starch enhances the tryptic digestion of protein in soybean (Boonvisut and Whitaker, 1976). However, factors influencing legume starch digestibility are elusive, and further precise research will be helpful in illustrating more information on legume crops.

The environmental effects are pronounced in legume starch; however, these have been rarely studied, and limited information has been available in soybeans and common bean. For instance, studies have been conducted on common bean (*Phaseolus vulgaris* L.) on how environmental conditions affect starch granule morphology, low-molecular-weight amylopectin, polymerization, average chain length, amylose, and digestibility of starch content within cultivating locations (Ovando-Martínez et al., 2011). Similarly, Dhungana et al. (2017a) reported environmental stability and correlation of seed starch in soybean with protein and oil contents. They observed significant G × E interactions for SSC. The evaluation of 3-year data suggested that high heredity values (95.5–96.6%) occurred due to genetics. However, the limited reports of environmental stability of seed starch content in legumes make it difficult to truly understand the trait behavior, and therefore more studies need to performed with different environments to understand the exact effect of allelic variation and environment for getting more stable starch content from legumes.

## Identified Mutants With Altered Starch Composition in Legumes

With the use of functional genomic approaches, several mutants with varied starch compositions have been characterized in *Arabidopsis* (Zeeman et al., 2010). For instance, *adg1*, *adg2*, and *pgm1* are recessive mutants of *Arabidopsis*, which is defective in starch anabolism, lacks the small subunit, and is called “starchless,” although they actually contain small amounts of starch in their chloroplasts (Lin et al., 1988; Ventriglia et al., 2008). Similarly, other mutants (*sus1/sus2/sus3/sus4*) have an absence of sucrose synthase (*SuSy*) activity in cell types except for the phloem. However, the mutant plants showed normal cell-wall structure and cellulose content, sugar content, lipid content, and seed weight and synthesized normal amounts of leaf starch (Barratt et al., 2009).

Legumes have wide habitats, their life cycles range from annual to perennial, and they vary in their genomes from simple diploid to polyploidy. Although sizeable genetic diversity is available in legume germplasms, diverse lines have been developed using artificial mutagenesis and breeding approaches. The first mutation studied at the molecular level in peas was the recessive mutation rugosus (*r*) (Bhattacharyya et al., 1990). Moreover, it was reported that genes at the *r* locus could offer further comprehension of mechanisms of seed development and seed composition. In pea, the number of mutants that affect various pathways of starch synthesis have been identified through breeding and mutation approaches. Decades before the role of pea starch, biosynthetic mutant genes at six loci (*r*, *rb*, *rug3*, *rug4*, *rug5*, and *lam* (*low amylose*)) was well established and functionally characterized for seed starch turnout, structure, and granular properties through mutagenesis approach (Lloyd et al., 1996; Craig et al., 1998; Bogracheva et al., 1999). Genes present at *r* (SBE I, SBEI), *rug5* (SSII), and *lam* (GBSSI) loci straightaway affect polymer biosynthesis and degree of starch branching. This alteration leads to an immense increase in amylose content of genotypes with an *r* and *rug5* loci from 60% to 75% and 43% to 52% in the total starch content, respectively. On the contrary, *lam* loci, which have been shown to lack SS activity, reduce the amylose content at 4–10%, which is significantly lower than in wild type on the basis of total starch (Boyer, 1985; Edwards et al., 1988), whereas substrate accessibility gets affected by *rb* [ADP-Glc pyrophosphorylase L1 subunit (AGPL1)], *rug3* (plastidial phosphoglucomutase (PGMP)), and *rug4* (sucrose synthase) loci. The other mutations in pea at “*rugosus*” loci are known to modify the shape of seeds from round to wrinkled and to exhibit pleiotropic effects on seed morphology (Bogracheva et al., 1999; Wang et al., 2003). Recently, Carpenter et al. (2017) studied the pea single plant accessions (*n* = 92) for association mapping. They observed that the natural allelic variation in *r*, *rb* (AGPL1), and *rug5* (SSII) was associated with chain length distribution (CLD) variation. From the reports, it indicates that the allelic difference at *r* locus has a great influence on pea phenotypes, which is associated with amylose content and amylopectin CLD that can produce the various forms of starch contents and structure *via* infusion of the genes at *r* and *rb* loci. Similarly, a study reported on another model legume species *Lotus japonicus* with a range of mutants for starch synthesis and accumulation. These isolated mutants are categorized as “synthesis mutants,” which are defective in a starch synthesis, which had a lower level of leaf starch, or breakdown “degradation mutants,” which had higher levels of leaf starch than do wild type. For instance, Vriet et al. (2010) identified mutations in phosphoglucoisomerase (*LjPGI1*), phosphoglucomutase, (*LjPGM1*), AGPase large subunit isoform (*LjAPL1* and *LjAPL2*), and Glc-1-P adenylyltransferase small subunit (*LjAPS1*), which lead to a huge reduction in starch contents or completely no starch due to lacking respective genes encoding enzymes. In addition, they evaluated L. *japonicus* population derived from the ethyl methanesulfonate (EMS) and further performed the mapping. They showed the relationship of AGPase and glucan water dikinase 1 (GWD1) enzyme with the starch-excess mutant phenotype. Moreover, the L. *japonicus* mutant studies focused on leaf starch content and information on other organs such as roots, embryos, and seeds. Generally, starch metabolism, physicochemical properties, and genetics are complex, and much less information is available across the legumes. Significant efforts in this regard have been made in some legume crops to improve seed carbohydrate composition through different approaches such as mutagenesis (Dobbels et al., 2017), quantitative trait locus (QTL) mapping (Kim et al., 2005; Dumont et al., 2009; Vandecasteele et al., 2011; Casañas et al., 2013), and marker-assisted selection (MAS) (Maughan et al., 2000). Such information needs to be utilized to understand gene interactions and their modes of actions in starch metabolism in various organs, especially seeds.

## The Genetic Basis of Starch Composition in Legumes

Recent advancements in sequencing technologies and genotyping platforms have facilitated the genetic dissection of the simple as well as complex agronomic traits across plant species (Kulkarni et al., 2018). Next-generation sequencing (NGS) technologies have become increasingly accessible and cost-effective, thereby providing an opportunity to reveal DNA variation through whole-genome sequencing of plants, which can be utilized in the genetic dissection of complex traits and evolutionary studies (Chapman et al., 2015; Kim and Buell, 2015). A very limited number of QTLs have been identified for the starch content in legume crops mentioned in **Table 2** (Casañas et al., 2013; Masari et al., 2017; Dhungana et al., 2017b). In addition, recently, Carpenter et al. (2017) performed association mapping in pea. Furthermore, they have identified polymorphisms in eight candidate genes from the pea seed carbohydrate and starch metabolic pathway, which have a significant association with variations in debranched or amylopectin chain CLD, and two genes that have a significant association with variations in amylose content. These genes are involved in substrate availability, chain elongation, and branching CLD.

**Table 2:** Previously reported QTLs/gene/SNP for seed starch/amylose contents or amylopectin length.

Crop	Trait	Mapping population/accessions	Chr/loci	PVE (%)	No. of QTLs/gene/SNP	Reference
Soybean	Seed starch content	Williams82 × PI 366121	6 and 15	5.6 to 11.3	9	Dhungana et al., 2017b
Common bean	Seed starch content	Xana × Cornell 49242/RIL	1, 2, 4, and 9	25	5	Casañas et al., 2013
Mung bean	Seed starch content	V6087AG 9 × V2050BY	8	12.3	1	Masari et al., 2017
Pea	Amylose	50 accessions	*Agpl1*,^c^ *Gbsts1*,^c^ and *Sbe2* ^c^	–	4^b^	Jha et al., 2015
	Total starch	50 accessions	*Sbe2*,^c^ *Sps*,^c^ and *SS* ^c^	–	10^b^	Jha et al., 2015
	Amylose	92 accessions	*r* ^c^	–	2^a^	Carpenter et al., 2017
	Amylopectin CLD	92 accessions	*r* ^c^	–	8^a^	Carpenter et al., 2017

a Gene.

b SNP.

c Loci.

CLD, chain length distribution; Agpl1, ADP-glucose pyrophosphorylase; Gbsts1, granule-bound starch synthase I; Sbe2, starch branching enzyme II; SPs, sucrose phosphate synthase; SS, second sucrose synthase; r, rugosus; Chr, chromosome; PVE, phenotypic variation explained; RIL, recombinant inbred line; QTL, quantitative trait locus; SNP, single-nucleotide polymorphism.

The SSC is a quantitatively inherited trait and is influenced by growing environments (Dhungana et al., 2017a). Very few QTLs for starch content are reported in legume crops (**Table 2**) compared with rice, maize, and other cereal crops. For instance, accession of mung bean V6087AG content with the highest SSC and through mapping a major QTL have been reported in mung bean (*qSSC8.1*) for SSC with 12.3% phenotypic variation explained (PVE) (Masari et al., 2017). They have reported high heritability of about 80% for SSC and high correction between the SSC and seed weight (*r* = 0.6). This suggested that lines with high seed weight may contain high starch content, so these can be used as an indirect selection method. The region identified in mung bean for high SSC further can be explored to develop improved starch content in mung bean through MAS. Similarly, Casañas et al. (2013) reported five QTLs for SSC with 11–15% of PVE in common bean. They used recombinant inbred lines (RILs) population for mapping and reported no significant differences between parents in seed contents. However, consistent transgressive segregations were significantly observed for amylose, apparent amylose, starch, ash, and uronic acid. From this study, it is indicated that QTL from seed coat components appears quite independent of seed cotyledon content. In soybean, Dhungana et al. (2017b) reported nine significant QTLs with 5.6–11.3% of the total PVE distributed over five different chromosomes in RIL derived from a cross of ‘Williams 82’ and ‘PI 366121’. The QTL *qSTR06_2* showed the highest PVE (9.1–11.3%). The reported results indicate that QTL has a significant environmental influence on the seed starch expression in the RIL population, and seed starch observed in the range of 0.11–1.39% showed continuous variation with transgressive segregation.

Breeding for the seed starch composition in legumes is a complicated process primarily due to the low variation in SSC. Hence, less focus has been given to identify loci, QTL, or genes identification for SSC. Moreover, it is necessary to identify the QTLs controlling the concentration of SSC using different genetic backgrounds across different environments. This can be simplified and accelerated using genomic resources, tools, and the dense genetic maps available for some of the legume species (Bandillo et al., 2015; Phansak et al., 2016).

## Transcriptome Advances in Legumes

Seed development is a complex process, and a precise understanding of the regulatory mechanisms involved in seed development is essential in order to explore the potentialities of improvement in seed compositions (glucose, sucrose, raffinose, and starch.). These processes involve transcriptional, metabolic, biochemical, and physiological reorganization by several different pathways and the associated changes in the expression level of a number of genes (Gutierrez et al., 2007; Santos-Mendoza et al., 2008). A combined proteomic and transcriptomic analysis of developing *Medicago truncatula* seeds showed differential expression of about 45% of the functionally classified seed-regulated genes during seed maturation (Gallardo et al., 2007). These genes were involved in other metabolic pathways such as carbohydrate, amino acid, lipid, energy, and secondary metabolism.

Recent transcriptome analyses have suggested a number of transcription factors (TFs) involved in seed development (Santos-Mendoza et al., 2008; Sreenivasulu and Wobus 2013). Liu et al. (2015) found that several soluble sugars and starch metabolism-related genes are significantly activated during the development of pea seeds, coinciding with the accumulation of sugars and starch in the seeds. Pradhan et al. (2014) carried out deep sequencing of transcriptomes from four seed developmental stages of chickpea and reported carbohydrate metabolism genes such as SS, debranching, starch cleavage, and galactinol synthase, highly expressed at late stages in comparison with 10 days after anthesis (DAA). On the other hand, higher expression observed for genes involved in the degradation of sucrose, starch, and metabolism of raffinose and trehalose at 10 DAA seed tissue as compared with 40 DAA. Similarly, Yang et al. (2015) performed a comparative genomic and transcriptome analysis in adzuki beans (*Vigna angularis*), in which they observed differentially expressed genes for starch and fat content in adzuki bean and soybean. They have reported 27 starch biosynthesis genes in adzuki bean and 46 in soybean; but no significant variation in the ratio of the starch biosynthesis genes has been observed according to the *χ* ^2^ test (*P* = 0.4135). However, the transcriptional level of starch biosynthesis and average transcription of individual starch biosynthesis genes in adzuki bean were significantly higher than in soybean at the mature seed stage. Such differences are thought to be caused by transcriptional abundance rather than copy number variations in the genes associated with starch and oil synthesis. Recently, phenotypic and deep sequencing of the transcriptome analysis performed in drought and salinity stressed chickpea by Garg et al. (2016) identified a number of genes involved in starch biosynthesis and UDP-glucose biosynthesis, which were induced by drought/salinity. In addition, starch safeguard and provide optimal energy to the stressed plants. Similarly, transcriptome study reported by the same group during seed development in two cultivars with contrasting seed size/weight (small seeded, Himchana 1 and large seeded, JGK 3) and observed a significant difference between the cultivars in the transcriptional level of the genes involved in starch biosynthesis. They found that more activity of genes involved in starch metabolism and photosynthesis in JGK 3 indicates that large seeds need more energy for cell division and maintain bigger seed size/weight (Garg et al., 2017).

With the use of NGS-based approaches, transcriptome analyses have been carried out to identify a number of genes involved in the regulation and function of seed development processes in plant species, for instance, *M. truncatula*, *Arabidopsis*, soybean, pea, chickpea, and adzuki bean (Girke et al., 2000; Gallardo et al., 2007; Jones et al., 2010; Severin et al., 2010; Jones and Vodkin, 2013; Pradhan et al., 2014; Liu et al., 2015; Yang et al., 2015). Large amounts of transcriptomic data have been generated in the recent past, and web portals exclusively for legumes are publicly available (**Table 3**). Research in this area may provide a blueprint of gene expression networks involved in the accumulation of nutrients and starch during legume seed development. Studies of this expression of genes may greatly help to improve the understanding of the molecular mechanisms behind the accumulation of several nutrients and starch during seed development.

**Table 3:** Web portals for transcriptome data of important legume crops.

Crop	Web portal	URL	Data type	Reference
*Cicer arietinum L.*	CTDB	http://www.nipgr.res.in/ctdb.html	RNA-seq	Garg et al., 2010
*Medicago truncatula, Glycine max, Lotus japonicus, Phaseolus vulgaris, Cicer arietinum and Cajanus cajan,*	LegumeIP	http://plantgrn.noble.org/LegumeIP/	RNA-seq and microarray	Li et al., 2015
*Lotus corniculatus*	LjGEA	http://ljgea.noble.org/v2/	Microarray	Verdier et al., 2013
*Lotus japonicus*	Lotus Base	https://lotus.au.dk.	RNA-seq and microarray	Mun et al., 2016
*Medicago truncatula*	MtGEA	http://mtgea.noble.org/v3/	Microarray	Benedito et al., 2008; He et al., 2009
*Arachis hypogaea L.*	PeanutDB	http://bioinfolab.muohio.edu/txid3818v1	RNA-seq	Duan et al., 2012
*Glycine max (L.) Merr.*	SoyPLEX	http://www.plexdb.org/plex.php?database=Soybean	Microarray	Dash et al., 2012
*SoySeq*	https://soybase.org/soyseq/	RNA-seq	Severin et al., 2010	

## Metabolomic Advances in Legumes

In legumes, a comparative study was carried out on the seed metabolome of pea lines with and without a major reserve protein, pea albumin-2, produced by deletion into a standard genetic background (Vigeolas et al., 2008). The deletion of this protein was linked with differences in amino acid and polyamine contents in the seed. Another study carried out a metabolic profiling of genetically modified and conventional soybean lines for 40 and 169 metabolites, respectively (García-Villalba et al., 2008; Clarke and Braun, 2013). However, the dynamics of primary photosynthate behavior between soybean and rice were compared by the researchers, who found that the carbon fluxes of photorespiratory and starch synthesis are high in soybean leaves (Nakamura et al., 1997; Okazaki et al., 2005; Matsuda et al., 2012). Recently, Das et al. (2017) performed an analysis of soybean leaf metabolites under controlled conditions, drought, and heat stress. They found metabolites for various cellular processes, such as glycolysis, the tricarboxylic acid (TCA) cycle, the pentose phosphate pathway, and starch biosynthesis. These metabolites involved in biosynthetic regulation of carbohydrate, amino acid, and peptide, purine, and pyrimidine metabolism. Recently, the nuclear magnetic resonance analysis of mature pea seed metabolites of three RIL populations reported by Ellis et al. (2018) showed the extensive genetic marker information and its association with loci and metabolite data. They observed significant variation within *r* or *rb* genotypes in the relative amount of amino acids, polyamine metabolism, sucrose-derived metabolites, secondary metabolites, and some unidentified compounds. This variation is controlled by multiple loci, and it affects the seed quality traits subsequently nutritional accept of seeds. Such metabolite variation could provide a basis for future seed component analysis.

Metabolomics is an emerging field, which can be a very useful tool to describe the association between phenotype and genotype. The metabolomic studies focusing on seeds are limited, and very few studies have been carried out at metabolomic levels for legume seed components. These studies have been limited to model species and mostly non-seed tissues. *Arabidopsis* has been a highly studied plant in aspects of seed metabolomics (Bottcher et al., 2008), followed by rice (Shu et al., 2008; Matsuda et al., 2012). However, reports of comprehensive metabolic profiling in legume crops are lacking. Advances in database development and bioinformatics tools are still lagging behind for legume seed metabolomics compared with the same in other crop systems. There are few database models, including ArMet (an architecture for metabolomics) designed for *Arabidopsis* and potato metabolomic studies (Jenkins et al., 2004). Similarly, an integrated database has been developed for soybean (Joshi et al., 2010) for mining and visualizing metabolomic data: SoyMetDB (http://soymetdb.org/). These tools, in addition to detailed experimental setups for desired metabolites, may be helpful for metabolic engineering efforts to enhance seed nutritional quality in soybean (Lin et al., 2014) and can be applied to other legumes to improve their nutritional and pharmaceutical values.

## Prospect for Starch Improvement in Legume Seeds

Legume starches are composed of about 13.6–88.0% amylose content shown in **Table 1**. However, the size, shape, and composition of legume starches vary with the amylose and amylopectin contents and the source, genotype, location, and physiological appearance of seed. In addition, high amylose starches impact thermal and viscosity properties and required higher temperature for processing and making different food items. The investigation reported on the thermal activity influenced by the amylopectin–amylose ratio as well as the amylopectin architecture (Chung et al., 2009; Copeland et al., 2009; Hoover et al., 2010). Furthermore, Joshi et al. (2013) evaluated the physicochemical properties of lentil starch and its impact on swelling, pasting, and gel formation activity and compared with those of corn and potato starches. They found that the highest amylose content is 32.5% in lentil among these starches. On the other hand, they observed that crystallinity and gelatinization enthalpy of lentil starch were the lowest. Recently, Carpenter et al. (2017) performed association mapping to identify the single-nucleotide polymorphisms (SNPs) and candidate genes for amylopectin CLD in the pea. Mutant alleles influence the gelatinization and pasting properties of starch. From the results, it indicates that presented pea lines containing the allelic variants can be used to explore for further studies of genetic control of pea seed starch structure and function. Moreover, a CLD factor in pea seed can serve as another useful approach to improve the structural and functional properties. Similarly, Edwards et al. (2018) reported the difference in the starch digestibility in wild-type and *r* mutant pea. Unlike other digestibility studies, which have focused on extracted starches, Edwards et al. (2018) used cooked macro-particles of pea cotyledons and extracted purified starch to define the influence of the *r* mutation on starch digestion kinetics in intact pea tissues. They observed that purified starches are more susceptible to α-amylase hydrolysis whereas encapsulated starch in integral plant cell is tolerant or more resistant to α-amylase hydrolysis. Moreover, considering the functional food and nutritional point of view, increasing the total amount of starch content in the mature seeds will be great interest in low starch-containing legumes such as soybean. In addition, specifically in other legumes, starch improvement will be highly desirable with an increase in amylose content, the ratio of amylose to amylopectin, and an increase in the RS content and slow digestible starch, which give immense nutritional and health benefits (Birt et al., 2013). Generally, the high amylose starch is indicative of improved RS content and SDS. This can lead to an increase in the endogenous level of legume RS by inbreeding of mutant allele or gene responsible for high amylose production and targeting the suppression of SBE enzymes.

From the literatures, it indicates that alterations in starch physicochemical properties have a substantial influence on the functional properties, which can be an incitement for modifying it efficiently. For instance, altering the amylose fraction can be useful in the product formulation for making more suitable gelled products, in addition to short and chewy bites, and can improve the crispiness of fried snacks and other food products. This may lead to the replacement of cereal and tuber starches, as it enhances the taste, gelling properties, and nutritional value of the product. However, information on legume starches is limited, so it is needed to study further precisely how to identify the proper amylose and amylopectin ratio, their structure, granule size, and physicochemical properties.

## Approaches for Starch Improvement in Legume Seeds

Globally, the current demand for starch and sugar from plants for various industrial applications is increasing. Although the major starches are isolated from cereals such as maize, rice, wheat, and sorghum, legumes may also play an important role in providing RS foods with high protein contents to humans. In legumes, starch accumulated during seed maturation varies among species (Zhao et al., 2018). An in-depth understanding of the basic process involved in the biosynthesis of harvesting seeds is required to modify the yield or quality of starches in legume plant species. Manipulation of the starch content in legume plants is achieved through the some basic strategies:

**“(1) Genetic and genomics method:** Advances in next-generation technologies contributed to the significant reductions in the sequencing costs, because of which millions of SNPs were discovered in crop genomes (Rasheed et al., 2017). In addition, genotyping by sequencing (GBS) and SNP genotyping arrays were developed for use in mapping studies of several legume crops, including soybean (SoySNP50K, 180K AXIOM^®^ SoyaSNP), cowpea (Illumina 60K iSelect BeadArray), peanut (58K Axiom_arachis SNP), pigeonpea (56K Axiom *Cajanus* SNP), and chickpea (50K Axiom^®^ *Cicer*SNP Array) (Song et al., 2013; Lee et al., 2015; Pandey et al., 2016; Roorkiwal et al., 2018; Saxena et al., 2018). This high-throughput technology enables genome-wide association study (GWAS), high-density genetic mapping, and novel allele and gene identification, which can be used for manipulating starch content-regulated trait variations according to prospected breeding needs. Moreover, reports indicate that these SSCs are correlated with genotypes evaluated, suggesting the interaction of the genetic loci that control them. Hence, it is necessary to identify the QTLs controlling the concentration of SSC using different genetic backgrounds across different environments. This high starch-containing genetic material can be used for developing improved cultivar for starch content.

**(2) Genetic engineering methods:** Potential biotechnological strategies have been reviewed in the past few decades (Frances and Bligh, 1999; Zeeman et al., 2010; Bahaji et al., 2014) such as the overexpression of BT1 proteins. This BT1 protein is also known as ADP-Glc or ADP antiporter found in the plastid internal membrane and plays an important role in regulating ADP-Glc flux into starch (Sakulsingharoj et al., 2004). Biotechnological methods, which have mostly demonstrated the modification of ADP-Glc pyrophosphorylase (*AGPase*) activity in plants, have been preferred to improve starch contents. The gene (*AGPase*) from *Escherichia coli* (*glgC*16) was transformed into potato plastids. The transgenic plants showed 60% more starch content in tubers than non-transgenic plants (Stark et al., 1992). However, this pattern was not consistent across other potato varieties (Sweetlove et al., 1996), and adverse effects have been reported, which contradict the initial study (Zeeman et al., 2010). This suggests that modification of the *AGPase* gene may not be an effective approach to improve the starch content in legume seeds. Down-regulation of a plastid adenylate kinase in potato plants was demonstrated to double the starch content and increase the yield of transgenic plants compared with wild-type plants (Regierer et al., 2002). Genetic engineering of starch-related genes such as *SS* I, II, III, and IV, *Sex4*, α-*amylase*, *GBSS*, glucan water dikinase, phosphoglucan, and water dikinase has been carried out in *Arabidopsis*, potato, and cereal crops. Additionally, this technological application has shown promise in enhancing the content of digestible carbohydrates in transgenic forage legumes such as clover (*Trifolium repens*) and alfalfa (*Medicago sativa*) (Frohberg et al., 2003). Manipulations have been carried out in pea through the generation of *rugosus* and *lam* mutants, which resulted in the modification of starch content, composition, and granule structure (Martin and Smith, 1995; Frances and Bligh, 1999). Further, this has been successfully applied to narbon beans (*Vicia narbonensis*), which are genetically altered for AGP-antisense inhibition (Rolletschek et al., 2002). The production of amylose content in legume probably can be increased by modification or inhibition in SBE enzyme-related pathways (Dupuis et al., 2014). In addition, common starch found in cereals (rice, corn, and sorghums) known as waxy starches (without amylose) are used in different food applications mainly as a thickening agent, preparation of nanoparticles, fillers and reinforcing agents in polymer composites or transporters for drug delivery, coating materials, and stabilizers (Evžen and Václav, 2017). This waxy or sticky type of starch can be produced by altering the GBSS enzyme pathway, and those legumes that are high in amylopectin production or with high CLD could be a useful target to produce waxy-type starch. However, increases in starch in the embryo or endosperm tissue in legumes are still elusive.

**(3) Mutation breeding:** The mutation causes sudden heritable change at the DNA level, which is not induced by genetic recombination and hybridization. Many mutants have been characterized in *Arabidopsis* that exhibit a starch excess phenotype and are mostly affected by enzymes involved in starch mobilization (Stitt and Zeeman, 2012). Mutation breeding is a powerful tool to generate a new genetic variation for quantity and quality of starch content. Through mutation breeding, it has a chance to discover mutant materials for quantity and quality of starch content and could be used as breeding materials to improve starch content in legume crops.

**Figure 2:**
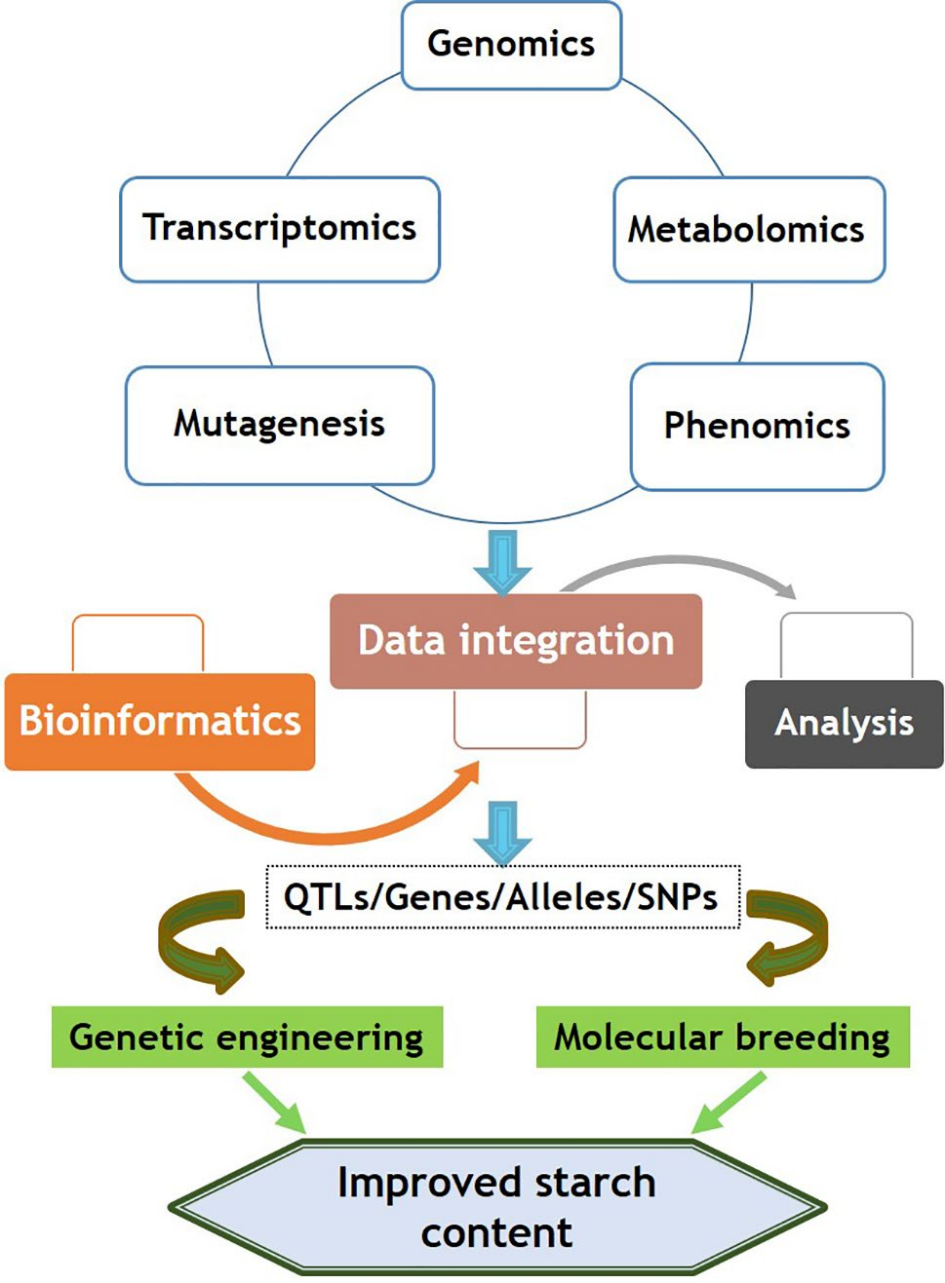
An integrated approach to improve seed starch content in legume crops.

The modification of starch quality and quantity in legumes is mainly affected by the number of genes involved in the process of synthesis, amylose and amylopectin ratio, their structure, granule size, physicochemical properties, and degradation. Hence, it is complicated to estimate what factors will be applicable for starch content improvement. In addition, a specific target to improve starch should be planned owing to its desirable application in industries. An integrated approach needed for starch improvement in legume crops is illustrated in **Figure 2**. This approach involves precise phenotyping of resources such as landraces, cultivars, and mutants; mutagenesis; molecular breeding; and knowledge of plant biology, genetic resources, bioinformatics, and biotechnological approaches in combination with conventional breeding techniques.

## Conclusion and Future Prospects

This review is focused on legume crops, which have significant potential benefits both for food processing and for human nutrition. Metabolism-related genes, enzymes, and their interaction involved in biological processes in starch and other carbohydrates need a systematic analysis and characterization. The identification of common factors controlling the expression of starch metabolism functions will be very vital to the design of molecular strategies aimed at improving starch content in heterotrophic organs of legume crops. Over the decades, conventional breeding strategies to increase SSC and increase digestibility have resulted in limited success. Very limited reports have shown stable and consistent QTL, genes, or SNP association with SSC. This limitation has occurred due to the complex nature of the trait. Although a number of QTLs and candidate genes have been identified in legumes, the dense genetic map is still only available for limited legume crops such as soybean, lentil, and peanut and is not consistently available across all legume crops. The structure and fraction of legume starches have not been precisely characterized as it in cereals. From the literatures, it indicates that there is lack of adequate information about structure of amylose and amylopectin of legumes starches. Furthermore, improvement of starch can be determined by targeting the ratio of amylose and amylopectin, CLD, RS, and SDS, which can influence the starch content and nutritional value. This can be achieved through major approaches by accessing the natural genetic variation and mutagenesis; using genetic modification of genes can exhibit a new phenotypic effect. However, it will be challenging to predict the kind of impact that genetic alteration imposes on the functional properties of legume starch and to improve legume starch for suitable use. The focus should be given to identify nutritionally improved and genetically diverse legume source and to characterize quality traits and its association with genetics. Recently, numerous advancements have been made in genetics, genomics, and the transcriptome area. However, the field of metabolites is still emerging rapidly and can be integrated with phenotyping and genomics to predict complex traits more precisely. Theoretically, the tools, genetic resources, and knowledge sources are currently available to develop enriched concentrations of the desired starch content in legumes, but there is significant scope to combine basic biological process with modern breeding technology. Further research into this area will address current difficulties and will help to develop a nutritionally rich and improved SSC in legumes crops. This will pave the way for breeder leading to select precise breeding material, which regulates consumer demand, enabling the execution of quality improvement of legume crops by breeding programs. In addition, it will create a new identity apart from being “poor man’s meat” with consumption of legumes for nutritional and health benefits.

## Author Contributions

RT wrote the manuscript. KK critically evaluated the manuscript for language. KK and HJ edited the manuscript. RT prepared illustrations, figures, tables, and references. JS and J-DL contributed critical comments to the draft. J-DL conceptualized, critically edited, and approved the manuscript. All the authors reviewed the draft.

## Funding

This work was supported by a grant from the Next-Generation BioGreen 21 Program (Project No. PJ01367401), Rural Development Administration, Republic of Korea.

## Conflict of Interest

The authors declare that the research was conducted in the absence of any commercial or financial relationships that could be construed as a potential conflict of interest.
